# Negative controls and how to use them: a guidance paper

**DOI:** 10.1093/ije/dyag163

**Published:** 2026-08-29

**Authors:** Sophie H Bots, Anna Schultze, Romin Pajouheshnia, Ian J Douglas, Rosa Gini, Olaf H Klungel, Rolf H H Groenwold

**Affiliations:** Division of Pharmacoepidemiology and Clinical Pharmacology, Utrecht Institute for Pharmaceutical Sciences, Utrecht University, Utrecht, The Netherlands; Faculty of Epidemiology and Population Health, London School of Hygiene & Tropical Medicine, London, United Kingdom; Department of Epidemiology, RTI-Health Solutions, Barcelona, Spain; Faculty of Epidemiology and Population Health, London School of Hygiene & Tropical Medicine, London, United Kingdom; ARS Toscana, Florence, Italy; Division of Pharmacoepidemiology and Clinical Pharmacology, Utrecht Institute for Pharmaceutical Sciences, Utrecht University, Utrecht, The Netherlands; Department of Clinical Epidemiology, Leiden University Medical Centre, Leiden, The Netherlands

**Keywords:** negative controls, confounding, bias, methodology

## Abstract

Negative controls have gained traction as a useful tool for causal research to address confounding and bias. However, general guidance on how to select and apply negative controls and how to report and interpret their findings is lacking. This paper lays out the key steps of conducting a negative-control analysis and provides all relevant background information, important feasibility and validity considerations, and practical suggestions for the implementation and interpretation of each step. This includes a convenient overview of all methods, complete with their rationale, key considerations, and illustrative examples, and practical checklists that both researchers and reviewers can use to facilitate the complete and rigorous reporting of negative-control analyses.

Key Messages

- Negative controls are variables that are not causally associated with the exposure (negative-control outcome) or outcome (negative-control exposure) of interest that share a similar bias structure with the association of interest and can be used to detect or control for bias.
- Negative-control frameworks primarily focus on (i) detection and correction and (ii) calibration of *P* values and confidence intervals, each making different assumptions.
- Researchers should pre-specify what they consider a null result and interpret the results of negative-control analyses accordingly.
- This article provides checklists to support the design and reporting of negative-control analyses, including the rationale, methods used, assumptions made, and limitations.

## Introduction

Negative controls have gained traction as a useful tool to identify and/or correct for (unmeasured) confounding [1, 2], selection bias [3], information bias due to misclassification [3, 4], and Type I error [5]. They have been used across epidemiological disciplines (Supplementary Table 1). However, the choice of a negative control is not straightforward and the analysis and interpretation of results are complicated by the existence of multiple approaches that each has its own assumptions [1, 5–7]. General guidance on how to apply, report, and evaluate negative controls is lacking [8, 9]. This paper provides guidance for researchers and readers on the key steps of negative-control analyses, from the selection of negative controls to reporting and interpretation of the results.

## The concept of negative controls

The reasoning behind negative controls can be traced back to the Bradford Hill criterion of specificity [10, 11] and the concept has since been applied under different names (Supplementary Table 1). The term itself was popularized in the field of epidemiology by Lipsitch and colleagues [1] referring to a sensitivity analysis to detect bias or confounding. They took inspiration from experimental researchers who often include test conditions that either neutralize or leave out a key ingredient of the exposure or include an outcome not affected by the treatment as a check because any association that arises under such a condition must be brought about by other factors that could distort the association of interest. Negative controls are thus divided into negative-control exposures (NCEs) and negative-control outcomes (NCOs) [1]. Translating this idea to epidemiology, they defined a negative control as a variable that (i) is not causally associated with the exposure (for an NCO) or the outcome (for an NCE) of interest and (ii) shares its bias structure with the exposure (for an NCE) or outcome (for an NCO) of interest [1].

In this framework, only a variable that meets both conditions can act as a valid negative control. For example, an NCO to detect (unmeasured) confounding should not be caused, directly or indirectly, by the exposure of interest [Condition (i)], but should share the bias structure (potential confounders) that could connect the exposure of interest with the NCO [Condition (ii)]. A variable that does not meet Condition (ii) (e.g. does not share its bias structure with the variable of interest) cannot serve as a negative control. Shi and colleagues have formalized these assumptions by using causal-inference notation [7].

Schuemie and colleagues introduced an alternative framework using negative controls for the calibration of *P* values [5] and confidence intervals [12] for Type I error. Within this framework, negative controls are defined as variables not causally associated with the exposure (for an NCO) or outcome (for an NCE) of interest; the shared bias structure is not explicitly considered when selecting negative controls. They instead propose that, with a sufficient number of negative controls, the distribution of the systematic error for the association of interest can be estimated and used for calibration.

## Rationale for using negative controls

Negative controls can be used for roughly two purposes: bias detection and bias correction. Table 1 and Supplementary Table 1 provide an overview of the available approaches per purpose, including their assumptions, practical guidance, and relevant examples.

**Table 1:** Overview of different aims of negative-control analyses, their key assumptions and considerations, and practical guidance on their implementation.

Purpose	Key assumptions and considerations
i. Bias detection	**Assumptions for all methods within this purpose** Negative controls are defined by following the Lipsitch framework Sources and expected direction of the bias should be defined a priori Confounding **Method-specific assumptions** The negative control is affected by the same (unmeasured) confounding as the variable (outcome or exposure) of interest **Literature:** see [1, 7] for illustrative directed acyclic graphs **Practical guidance** Create an overview of all common causes of the variable of interest and identify the relevant unmeasured confounders of the variable of interest that the negative control is meant to detect (the bias structure). Drawing a directed acyclic graph can support this process Compile a list of potential negative controls, starting with variables that at least meet Condition (i): no causal association with the variable of interest Strategy 1: manually identify potential negative controls following the approach outlined by two papers trying to compile a reference set of NCEs for four relevant drug-safety outcomes [23, 24]. They list a few requirements that a good NCE should adhere to (that also apply to NCOs), which read like an operationalization of the Bradford Hill criteria. The requirements are: (1) the outcome should not be listed as an adverse reaction in the product label, (2) the medication should not be listed as a causative agent in literature on drug-induced diseases, (3) randomized–controlled trials and large observational studies should have failed to demonstrate an association (relative risk > 1), (4) there should be no definitive or highly suggestive case reports linking the medication and the outcome, (5) there should be no analogous medication–outcome pairs, (6) there should be no experimental evidence (e.g. animal studies) of an association. Importantly, this evidence should have accrued over a sufficiently long time and dose range based on sufficiently sized studies. The authors searched medication product labels, relevant textbooks, and published literature to identify medications that met these criteria Strategy 2: use an automated screening system to identify potential negative controls. Feed the relevant information sources (medication product labels, textbooks, published literature) that you have collected through a screening system that will automatically identify positive and negative controls. An example is the LAERTES program developed by Voss and colleagues [25] Strategy 3: search for existing studies that applied one (or more) negative control(s) within the same or a highly similar context of your study Manually check whether the variables on the preliminary list also meet Condition (ii): sharing relevant measured and unmeasured confounders with the variable of interest (e.g. a shared bias structure). This can be done by using published literature, relevant textbooks, and reliable medical websites that describe either the main indications (in the case of an exposure) or risk factors (in the case of an outcome) for each variable Select the negative control(s) that you will use in your analyses. This may be relatively straightforward if you find a negative control that perfectly meets both Conditions (i) and (ii). Often, however, a negative control may have a similar but not completely identical set of confounders to the variable of interest. It is good practice to consider beforehand what you will consider “identical”: does the negative control need to share all confounders or would a number of key (unmeasured) confounders be sufficient? Similarly, does the effect of the confounder need to be of the same magnitude or is it sufficient if the direction of the effect is the same and the magnitude is within a certain range? We recommend writing these considerations down in your analysis protocol for optimal transparency (see STROBE-style checklist in Supplementary Table 2) Repeat your main analyses but substitute the variable of interest with the negative control(s) Report and interpret the findings (see STROBE-style checklist in Supplementary Table 2) Selection bias **Method-specific assumptions** Selection of participants into a study based on the negative control is affected by the same degree of selection bias as when the variable (outcome or exposure) of interest would be used **Literature:** see [3] for illustrative directed acyclic graphs **Practical guidance** Create an overview of all factors influencing the variable of interest and identify the relevant selection-bias factors of the variable of interest that the negative control is meant to detect (the bias structure). Drawing a directed acyclic graph can support this process Compile a list of potential negative controls, starting with variables that at least meet Condition (i): no causal association with the variable of interest. You can follow the strategies outlined under confounding Manually check whether the variables on the preliminary list also meet Condition (ii): sharing relevant selection-bias factors with the variable of interest (e.g. a shared bias structure). The bias structure is likely to be unique for each study, as it is heavily influenced by design choices and context. We recommend drawing directed acyclic graphs for high-potential negative-control candidates to visualize whether their bias structure matches that of the variable of interest Select the negative control(s) that you will use in your analyses, following the approach described under confounding Repeat your main analyses but substitute the variable of interest with the negative control(s) Report and interpret the findings (see STROBE-style checklist in Supplementary Table 2) Information bias (specifically differential measurement error) **Method-specific assumptions** The negative control shares a common source of measurement error with the variable (outcome or exposure) of interest **Literature:** see [3] for illustrative directed acyclic graphs **Practical guidance** Create an overview of all factors influencing the variable of interest and identify the relevant information-bias factors of the variable of interest that the negative control is meant to detect (the bias structure). Drawing a directed acyclic graph can support this process Compile a list of potential negative controls, starting with variables that at least meet Condition (i): no causal association with the variable of interest. You can follow the strategies outlined under confounding Manually check whether the variables on the preliminary list also meet Condition (ii): sharing relevant information-bias factors with the variable of interest (e.g. a shared bias structure). The bias structure is likely to be unique for each study, as it is heavily influenced by design choices and context. We recommend drawing directed acyclic graphs for high-potential negative-control candidates to visualize whether their bias structure matches that of the variable of interest Select the negative control(s) that you will use in your analyses, following the approach described under confounding Repeat your main analyses but substitute the variable of interest with the negative control(s) Report and interpret the findings (see STROBE-style checklist in Supplementary Table 2)
ii. Bias correction	**(a) Risk-estimate correction** Control outcome calibration approach (COCA) (corrects risk estimate for unobserved confounding using an NCO) **Rationale:** assume that there is no association between the NCO and the exposure of interest if we correct for all confounders (measured and unmeasured). Given that an NCO has no causal association with the exposure of interest, all (potential) outcomes for the NCO (observed or counterfactual) should be independent of the actual exposure status. If this is not the case, then we have not corrected for all relevant confounders. For additive causal effects, the magnitude of unmeasured confounding can be estimated by using the following linear regression model: *Z = β*_0_ *+ β*_1_ *C + β*_2_ *Y*(*ψ) + β*_3_ *X*, where *Y*(*ψ*) is a measure that combines the observed outcome and possible values of the unmeasured confounding (*ψ*). By alternating values for *ψ*, one can look for a situation in which *β*_3_ = 0 (i.e. NCO independent of exposure) and thus obtain a measure for unmeasured confounding (*ψ*). **Method-specific assumptions** Negative controls are defined by following the Lipsitch framework Rank preservation of individual’s counterfactuals under treatment versus control conditions, i.e. constant additive individual causal effect **Literature:** see [17] for detailed rationale, mathematical derivation, and an example; see [6] for discussion on caveats **Practical guidance** Create an overview of all common causes of the variable of interest and identify the relevant unmeasured confounders of the variable of interest that the negative control is meant to detect (the bias structure). Drawing a directed acyclic graph can support this process Search for existing studies that applied one (or more) negative control(s) within the same or a highly similar context of your study. As the COCA specifically requires one NCO that meets both Conditions (i) (no causal association) and (ii) (shared measured and unmeasured confounders), it is most efficient to search for NCOs that have already been used and have therefore (presumably) been quality-checked by other researchers. If there are no previously published articles, then you can switch to the other strategies listed under the confounding section of the bias-detection row above Select the NCO that you will use in your analyses, following the approach described under confounding Compute both the crude effect of the exposure on the outcome and the adjusted effect through applying the COCA. The adjusted effect is called the COCA estimator. The exact calculation depends on whether you have a continuous or dichotomous outcome, following [17] Statistically test whether the crude effect and the COCA estimator are different from each other. For continuous outcomes, this is done via the Hausman test: calculate a confidence interval for the difference between both estimators. Under the null hypothesis of no confounding, this interval should include 0. For dichotomous outcomes, either the delta method or nonparametric bootstrap can be used to calculate the confidence interval for the difference Report and interpret the findings (see STROBE-style checklist in Supplementary Table 2) Confounding bridge or double-negative-control approach (corrects risk estimates for unobserved confounding using both a NCE and an NCO) **Rationale:** assume that the confounding affecting an NCO matches the magnitude and direction of confounding present in the association between the exposure and outcome of interest. There exists a mathematical transformation for the effect of the exposure of interest on an NCO that captures the influence of confounding, which is called the outcome confounding bridge. The essence of this method is the assumption that the confounding effect of *U* on *Y* at a certain level *x* of *X* is equal to the confounding effect of *U* on the transformation of *Z* and *x*, denoted by *b*(*Z*, *x*) In other words: *E*(*Y*|*U*, *X* = *x*) = *E*[*b*(*Z*, *x*)|*U*, *X* = *x*] This equation can be solved with an NCE (*W*) because there should not be an association between an NCE and either *Y* or *Z* if there is no unmeasured confounding *U*. In this way, the influence of confounding can be estimated and controlled for in the analysis for the association of interest **Method-specific assumptions** Negative controls are defined by following the Lipsitch framework The outcome confounding bridge function exists Completeness: if solving the confounding bridge with the NCE gives a null result (e.g. no unmeasured confounding), then we can assume that there is no measured confounding for any level of the (negative-control) exposure **Literature:** see [18, 30] for detailed rationale, mathematical derivation, and practical implementation; see [6] for discussion on caveats **Practical guidance** Create an overview of all common causes of the association of interest and identify the relevant unmeasured confounders of the association of interest that the negative control is meant to detect (the bias structure). As the confounding bridge requires both an NCO and an NCE, relevant confounders for both the outcome and the exposure must be identified. Drawing a directed acyclic graph can support this process Search for existing studies that applied one (or more) negative control(s) within the same or a highly similar context of your study. As the confounding bridge specifically requires one NCO and one NCE that meet both Conditions (i) (no causal association) and (ii) (shared measured and unmeasured confounders), it is most efficient to search for negative controls that have already been used and have therefore (presumably) been quality-checked by other researchers. If there are no previously published articles, then you can switch to the other strategies listed under the confounding section of the bias-detection row above Select the NCO and NCE that you will use in your analyses, following the approach described under confounding Specify the confounding bridge equation using both the NCO and the NCE. Due to the fact that the NCO and NCE can only be connected through shared unmeasured confounders, the bridge equation becomes two linear equations with two unknown parameters that can be solved. It is known as the Fredholm integral equation of the first kind [18]. It is recommended to specify the confounding bridge model as either linear additive or exponential multiplicative, although more flexible approaches can be used if you worry that the simpler models are at risk of model misspecification Solve the confounding bridge equation and evaluate whether unmeasured confounding is present (see STROBE-style checklist in Supplementary Table 2 for suggestions on how to define a priori thresholds for this) If the confounding bridge suggests that unmeasured confounding is present, rerun the original analysis, but incorporate the negative controls to adjust for the unmeasured confounding. See [18] for the exact steps and equations Report and interpret the findings (see STROBE-style checklist in Supplementary Table 2) **(b) *P*-value and confidence-interval calibration** *P*-value calibration (calibrates *P* values for Type I error—the error of falsely rejecting the null hypothesis) **Rationale:** instead of relying on a hypothetical null distribution for hypothesis testing, negative-control estimates can be used to derive an empirical null distribution against which to test hypotheses. The empirical null distribution is an approximation of the bias distribution underlying the association of interest; each negative-control estimate can be considered as a sample of that distribution. Thus, if there is bias in the association of interest, then the mean of the empirical null distribution will not be zero, and *P* values should be calibrated accordingly **Assumptions** Negative controls are defined by following the Schuemie framework The bias underlying the association of interest follows a normal distribution **Literature:** see [5] for detailed rationale, mathematical derivation, and practical implementation **Practical guidance** Compile a list of potential negative controls that at least meet Condition (i): no causal association with the variable of interest. Although the original publication preferred NCEs over NCOs, as the authors expected exposure measurement to be more reliable [5], both have been applied within this context (see Supplementary Table 1). This method requires a larger number of negative controls than previous ones because it tries to derive the empirical null distribution. The original publication used 37 and 67 NCEs for the two use cases that they provided, respectively, which the authors identified through manual screening following Strategy 1 outlined under the bias-detection confounding section [5]. Using an automated screening tool or existing negative-control reference sets may be more efficient and could be preferred if available and feasible Create the empirical null distribution by fitting a Gaussian probability to the negative-control estimates, taking into account the sampling error of each estimate. The original publication provides analysis code (R code) for this in their supporting information (Appendix D) [5] Calculate the calibrated *P* value by using the mean and standard error of the empirical null distribution. The original publication also provides R code for this [5] Report and interpret the findings (see STROBE-style checklist in Supplementary Table 2) Confidence-interval calibration (calibrates confidence intervals for Type I error—an extension of the *P*-value calibration method) **Rationale:** same as the *P*-value calibration method **Assumptions** Same as the *P*-value calibration method Synthetic positive controls can be constructed by multiplying the risk found for negative controls. In other words, assuming that the negative control has an effect estimate of 1, by doubling the incidence of the NCOs in the dataset, a synthetic positive control will be created with an effect estimate of 2. This requires the assumption that each synthetic outcome has the same measurement error as the corresponding negative outcome [31] **Literature:** see [12] for detailed rationale, mathematical derivation, and practical implementation **Practical guidance** Compile a list of potential negative controls that at least meet Condition (i): no causal association with the variable of interest. The original publication used 50 NCOs that they selected through the automated screening method followed by manual review based on the prevalence of each NCO in the database [12] Create a set of positive controls by modifying the prevalence of selected NCOs using predictive modeling (*L*_1_ regularized survival regression). Choose one or more target prevalence rates that you want the positive controls to have and report these in the methods section Creating the empirical null distribution and calculating the calibrated confidence interval follows similar logic to the *P*-value calibration, but the calculations now involve both the positive and negative control estimates and sampling errors. Analytical code for both is available in the form of an R package called EmpiricalCalibration, which does require the data to be in OMOP format [12] Report and interpret the findings (see STROBE-style checklist in Supplementary Table 2)

First, negative controls can be used to detect bias as a sensitivity analysis to test the robustness of study findings—an approach that seems to be widely supported across the epidemiological community and fits within the triangulation framework [13]. This approach follows the negative-control definition of the Lipsitch framework, discussed in the previous section. Additionally, the direction of the bias should be defined a priori, so that it is clear which hypothesized bias mechanism is being tested [13, 14]. The assumption of a shared bias structure is untestable, although methods have been proposed [15], and finding a negative control that perfectly meets both conditions of the Lipsitch framework is difficult [16].

Second, negative controls can be used for bias correction through two different approaches. The first approach corrects effect estimates for a specific, a priori defined bias. Methods within this approach build upon the Lipsitch framework, but require additional assumptions regarding the bias structure [17, 18] (Table 1). Violation of these assumptions may threaten the validity of the correction [6, 9]. In addition, a simulation study has shown that correcting estimates for bias by using NCEs is not reliable when there is (differential) measurement error in either the exposure of interest or the negative control itself [19]. The second approach calibrates *P* values and confidence intervals [5, 12] to account for uncertainty due to the systematic overestimation of effects (Type I error) present in observational studies in general. Methods within this approach follow the negative-control definition of the Schuemie framework, relying on the key assumption that a large-enough pool of negative controls will represent the full range of biases relevant to the association of interest [16, 20, 21]. For the *P*-value method, the calibrated *P* values are not more reliable than uncalibrated values when this assumption is violated [20]. Moreover, calibration works best in situations in which unmeasured confounding is the main concern and the negative controls share the bias structure of the variable of interest [22]. In addition, calibrating *P* values decreases the risk of Type I error at the cost of increasing the risk of Type II error [20, 21]. One additional practical consideration is that the calibration of confidence intervals also requires the addition of synthetic positive controls to the dataset [12].

## Selection of negative controls

This section outlines a step-by-step approach to selecting negative controls, as summarized in Supplementary Table 2 (Part 1). Researchers may need to cycle through these steps before finalizing their negative-control selection and analysis plan.

The first step is deciding the goal of the negative-control analysis: identifying what the anticipated sources of bias are and how they could be addressed through detection or correction. The second step is compiling a list of potential negative-control candidates that are not causally related to the exposure (NCO) or outcome (NCE) of interest. These could be identified based on subject knowledge or previous studies with similar associations of interest, selected from published lists of negative controls [23, 24] or identified via an automated database search [25] ideally coupled with a manual review of the suggested candidates [16]. The third step is assessing the value of these candidates on underlying assumptions of the negative-control purpose(s) and data requirements.

There may also be feasibility concerns. First, negative-control analyses for bias detection generally repeat the main analysis while replacing the variable of interest with the negative control, which has practical implications for some study designs. For example, as a case–control study samples the study population based on the presence/absence of the outcome, using an NCO would require two separate study populations, whereas an analysis of an NCE could be performed within the original sample. The prevalence of a negative control may limit feasibility, as rare NCOs and NCEs may have insufficient power [7]. The (expected) validity of the negative-control measurement is contingent upon any design-specific assumptions. For example, negative controls used within a self-controlled design should meet the design-specific assumption that neither the exposure nor the outcome is chronic. Feasibility concerns such as measurement quality and presence in the dataset should be considered. Second, some bias-correction approaches specifically require either an NCE or an NCO or both [9], so these cannot be applied if a suitable candidate cannot be found. Importantly, bias-correction approaches typically require additional analysis steps (Table 1 and Supplementary Table 1).

## Reporting negative-control analyses

A recent review of 184 papers using negative controls showed that 43.5% did not specify the type of bias that they tried to address and only 4.3% explained their reasoning behind assuming a shared bias structure between the negative control and the variable of interest [8]. This hinders reviewers and readers in appraising whether the negative-control analysis was appropriate and valid. It is important to report the reason for performing a negative-control analysis and the rationale behind choosing a specific (number of) negative control(s). Other details depend on the goal of the analysis, as the practical implementation differs across approaches. It is also important to pre-specify what authors consider to be a null result, how this will be interpreted, and whether the power of the negative-control analysis was considered beforehand.

Correction and calibration approaches require more extensive reporting. Bias-correction approaches often rely on specific assumptions (Table 1) to give valid results [6]. Any assumptions should be verified (when possible) and reported. Calibration approaches use the selected negative controls to construct the underlying error distribution and then calibrate either the *P* value or the confidence interval—an analysis step that should be reported separately. Table 1 provides practical guidance and additional literature on the implementation of these approaches.

Supplementary Table 2 (Part 2) provides a reporting checklist, which can double as a critical appraisal tool.

## Inference based on negative controls

The main assumption underlying negative controls is that there should be no association between the negative control and the variable of interest in the absence of bias [1]. Under ideal conditions and strong assumptions, a null finding signals the absence of bias, while a non-null finding signals the presence of bias. In reality, negative controls may lack power and thus show a null finding due to imprecision of the estimate, two sources of bias with opposing directions may result in a null finding [1], or there may be suboptimal overlap of the bias structure between the negative control and the exposure or outcome of interest. Researchers should pre-specify what they will consider a null finding in a negative-control analysis [26]. Supplementary Table 2 (Part 1) provides an overview of possible options.

Negative controls are, in some instances, poor at detecting biases that move estimates toward the null, such as misclassification [27]. Positive controls may be a more effective tool for those situations.

As mentioned earlier, methods within the Lipsitch framework share the important but unverifiable assumption that the variable of interest and the negative control share their bias structure [1, 14]. Precisely because this assumption cannot be verified, it is generally recommended to appraise the outcome of a negative-control analysis as part of the totality of evidence [11, 13]. Whether or not the found association of interest can indeed be interpreted causally can then be supported by a negative-control null finding, especially if other sensitivity analyses point in the same direction and vice versa. This interpretation should also consider the confidence in how strongly the negative control shares the bias structure and how deviations might affect the conclusions. For example, if the negative control misses one relevant common cause, then a null result does not support a total absence of bias [1]. For bias-correction methods, the degree to which method-specific assumptions were met should also be taken into account when interpreting the results [6].

Within the Schuemie framework, an association with a low level of bias will have a very narrow underlying bias distribution centered on the null. The crucial, but also unverifiable, assumption here is that the set of negative controls properly reflects the bias present in the association of interest [16], which should be considered when interpreting the results. The calibration of *P* values or confidence intervals to reduce the risk of Type I error is less effective when the negative controls do not properly reflect the true underlying bias [22]. In addition, calibration increases the risk of Type II error [21], especially in the presence of measurement error [20], because it inflates the *P* values and confidence intervals to incorporate the uncertainty detected by the negative controls. Calibrated values should be compared to the original values to assess which are the most reliable given the potential caveats of the calibration method.

## Further remarks

In their simplest interpretation, negative controls can be used to detect problems in an analysis, such as model misspecification (Supplementary Table 1). However, they can also be combined with other sensitivity analyses as part of the triangulation effort within or across studies. The application of multiple negative controls for bias detection warrants further consideration [9]. It is unclear whether using multiple negative controls has added benefit and how the findings should be summarized and interpreted. Multiple negative controls that point toward the same conclusion may strengthen the bias-detection analysis. Conversely, multiple negative controls that point in different directions will complicate interpretation of the findings. In addition, meta-analysing the findings in such a situation may lead to an overall estimate of no bias, even though individual estimates may suggest that bias exists. One suggestion is to rank negative controls on their likelihood of meeting relevant assumptions, creating an a priori hierarchy of reliability to inform interpretation.

Bias-correction approaches have been extended to empirical evaluation or the benchmarking of study designs and a priori testing of data and design suitability to answer a specific research question (Supplementary Table 1). This requires researchers to define a priori the way in which the results of the negative-control analysis will be interpreted and informs decisions around study design at the protocol stage. In cases in which the findings suggest that the research is infeasible, resources can be dedicated elsewhere. However, inherent limitations of the negative-control analysis (violation of assumptions, measurement error) could lead to erroneous conclusions about the data or design suitability, leading to studies not being conducted when they could have yielded useful information. If negative-control analyses are used to rule out a design or data source, then the assessment and conclusions should be published (in the protocol or study report), to permit external critical appraisal of the decisions.

Negative controls have also been mentioned in the growing literature on proximal causal inference. Proximal causal inference questions whether the exchangeability assumption is ever truly met in observational studies because covariates are often measured with error and therefore are at best proxies of the true underlying values and associations [28]. In this context, negative controls are seen as proxies for unmeasured confounding and bias-correction methods such as the confounding bridge can be applied to leverage these proxies for causal inference [18, 28].

Importantly, the results of negative-control analyses are attributed a causal meaning, as we assume that (through the shared bias structure) they capture the true effect plus any bias that exists. Therefore, all identifiability assumptions laid out by the causal-inference framework (i.e. exchangeability, consistency, positivity) should be considered when selecting negative controls. Given this context, there has been growing interest in applying negative controls within the target trial-emulation framework. The framework encourages the use of active comparators, which are considered a special kind of NCE (Supplementary Table 1). NCOs could also be applied within this framework as a sensitivity analyses. However, the framework has also been criticized [29], suggesting that both its application and the incorporation of negative controls may be most valuable for studies focusing on individual-level exposures.

## Conclusion

Negative controls are an analytical tool to detect or correct bias and can be applied in different ways. This article provides an overview of current methods and their limitations together with practical checklists to support the design, reporting, and critical appraisal of such analyses.

## Ethics approval

This work did not involve animal or human studies and thus required no ethics approval.

## Supplementary Material

dyag163_Supplementary_Data

## Data Availability

No new data were generated or analysed in support of this research.
