# Supplementary material for: Negative controls and how to use them: a guidance paper
Source: Int J Epidemiol. 2026 Aug 29;55(5):dyag163. doi: 10.1093/ije/dyag163 (PMC13525841; doi:10.1093/ije/dyag163)
Supplement: Supplementary file 1 [file dyag163_supplementary_data.zip › ije-2025-06-1048-File003.docx]

Supplementary Table S1	Overview of negative control applications across epidemiological disciplines

| **Discipline** | **Short description of negative control methods, terminology, and applications** |
| --- | --- |
| Pharmaco-epidemiology | Pharmaco-epidemiology concerns itself with specific treatments (vaccines, medications, medical devices) and their related effectiveness and safety outcomes. As such, factors that influence the likelihood of both the treatment and outcome of interest, like confounding by indication or immortal time bias, are main concerns [1]. In addition, it seems that negative controls have been adopted quite enthusiastically by this field, with a recent review identifying 184 pharmaco-epidemiology papers that applied some kind of negative control method [2]. The majority (81%) used the negative control method to detect bias, most commonly unmeasured confounding (51%). Negative control outcomes (50%) were used more often than negative control exposures (29%) or combination methods (19%).  Bias detection  Inactive comparators, defined as a medication (class) known not to be (causally) associated with the outcome of interest, that are used in a similar way to the investigational treatment (e.g., shared bias structure), can be considered negative control exposures to detect time-related biases [3]. For example, a dose of nasal corticosteroids too low to plausibly have a systemic effect has been used to check whether the observed risk reduction of nasal corticosteroids on asthma occurrence may be due to immortal time bias (selection bias) [4].  There are many examples of negative control outcomes in pharmaco-epidemiological literature [2], but a recent example used otitis externa as a negative control outcome for myocarditis to check whether the observed increased risk of myocarditis due to COVID-19 vaccines may be (partially) the result of confounding [5]. The authors outlined the main risk factors for the outcome that could act as confounders and tried to identify outcomes that shared these or similar risk factors without having been linked to COVID-19 vaccines.  Bias correction  Active comparators, defined as a medication (class) with similar indication and formulation as the treatment of interest but no causal effect on the outcome of interest, can be considered negative control exposures that specifically target confounding by indication [3]. Given that active comparators serve as the comparator group and thus automatically adjust the analysis for any confounding by indication that may be present, analyses using active comparators can be considered a bias correction approach [6].  Londhe and colleagues applied a variety of negative control strategies to contextualise their findings regarding the association between several exposures and the risk of retinal detachment. This included the use of negative control exposures, p-value calibration, and the risk of retinal detachment in a period preceding exposure [7]. More examples of bias correction applications can be found in a recent review [2]. |
| Clinical epidemiology | Clinical epidemiologists may refer to negative control outcomes as falsification endpoints [8-10], although both terms are used. The application of negative controls within a clinical context is otherwise similar to the pharmaco-epidemiological context.  Bias detection  Hamer and colleagues used mortality due to accidents as a negative control outcome to detect unmeasured confounding in the association between physical activity and cardiovascular mortality, and included a comparison of risk factors between the negative control outcome and the outcome of interest to evaluate whether they indeed shared common causes [11]. Zaadstra and colleagues used the negative control exposures (which they call “probe variables”) concussion, tonsillectomy, and broken arm to detect recall bias in a case-control study on the association between infectious mononucleosis and multiple sclerosis [12].  Bias correction  McQuade and colleagues calibrated their effect estimates and 95% confidence intervals when studying the effect of natural infection by common enteric pathogens on risk of diarrhoea in addition to using pathogens of the same type as negative control exposures [13]. |
| Clinical trials | It has been pointed out that placebo treatment in general can be considered a negative control exposure since the active ingredient is left out of the exposure. However, the placebo is subject to the same bias structure as the active treatment under study [14]. In addition, while proper clinical trial design, conduct and analysis protects against confounding and bias, there are many instances in which their validity is still threatened by some type of bias or confounding that could be addressed through negative control outcomes [14].  Bias detection  The assumptions for using either assignment to placebo (*intention-to-treat*) or placebo adherence (*per protocol*) as a negative control exposure have been formalised previously [15,16], and are summarised below:   - treatment assignment is random (to ensure exchangeability) - the placebo is *U-comparable* to the active treatment - the counterfactual outcome in the treatment arm is either exactly zero (*intention-to-treat* for outcome at end of follow-up) or on average equal to the average observed outcome in the placebo arm at the start of follow-up (*intention-to-treat* for changes since baseline). - placebo adherence has no effect on the outcome (for *per protocol*)   Negative control outcomes can be of value in situations where randomisation is broken because treatment groups are re-assigned, as in *as treated* and *per protocol* analyses [14]. They can also be used when differential outcome misclassification (measurement bias) is a concern [14]. |
| Environmental epidemiology | There is an alternative approach to negative controls that is often applied in the context of environmental exposures like air pollution, namely time-based methods [17-19]. Generally, time-based negative control definitions consider a moment or period in time where the causal effect is (expected to be) absent, but bias structure (e.g., unmeasured confounders) are similar to the association of interest.  Bias detection  It seems that negative controls for bias detection are rarely if ever used in environmental studies [20]. This may be because it is difficult to find negative control alternatives for population-level exposures or outcomes affected by population-level exposures that are not time-based. However, it has been argued that there is opportunity to apply negative control outcomes more within this field, for example using deaths from violence as a negative control outcome in a study looking at exposure to aerial pesticides [20].  Bias correction  Time-based methods predominantly focus on bias correction. Importantly, unlike other negative control methods, time-based methods incorporate the negative control variable in their regression models instead of re-running the original analysis with the negative control instead of the variable of interest.  Sofer and colleagues have shown that the difference-in-differences (DID) approach can be leveraged for negative control analyses [18]. In short, the DID approach corrects a treatment effect for unmeasured confounding by regressing the change in risk of the outcome over time (pre- and post-exposure) on the exposure. Given that the outcome cannot be affected by subsequent exposure, and that it is affected by the same set of confounders as the outcome after exposure, the pre-exposure outcome can serve as a negative control outcome. Using similar reasoning, post-outcome exposure can be considered a negative control exposure, and two methods to incorporate this into regression models have been proposed [17,19].  This does not mean that only time-based methods are used within this discipline. For example, Wang and colleagues applied the double negative control approach in their study to evaluate the effect of long-term exposure to low concentrations of air pollution and cardiovascular disease [21]. |
| Occupational epidemiology | Negative controls have been mentioned in the discourse around assessing risk of bias in occupational epidemiology [22], but actual examples are scarce. This may be due the fact that in occupational epidemiology, like in environmental epidemiology, there is a tendency to evaluate population- or group-level exposures, which complicates identification of negative controls [20].  Bias correction  Richardson and colleagues have proposed a method that uses a negative control outcome to adjust the standardised mortality ratio when the mortality rate in the external reference population is different from that in the study cohort due to non-comparability of both populations [23]. |
| Perinatal epidemiology | Studies evaluating the effect of pre-natal exposures on disease risk later in life share the unique advantage that they can use paternal exposure as a negative control exposure.  Bias detection  Brew and colleagues proposed a causal model for using paternal exposure as a tool to detect unmeasured confounding, complete with the relevant causal diagram and a worked-out example evaluating the effect of maternal distress on offspring asthma risk, with paternal distress as the negative control exposure [24]. Other authors used paternal smoking as a negative control exposure for maternal smoking in the context of offspring birthweight [25], or paternal substance use as a negative control for maternal substance use in the context of offspring attention deficit hyperactivity disorder [26].  Alternatively, time-based definitions like maternal exposure before pregnancy can be used. For example, Brynge and colleagues used maternal infection in the year before pregnancy as a negative control exposure when evaluating the effect of maternal infection during pregnancy on diagnoses of autism and intellectual disability in the offspring [27]. |
| Mendelian randomisation | Mendelian randomisation (MR) assumes that the genetic variant under study is only associated with the outcome of interest through the exposure. This assumption is violated when the way a population is structured affects both the distribution of the genetic variant and the outcome within that population, introducing a spurious association between the two. This is called population stratification, and it is the one of the main concerns in MR studies [28].  Bias detection  Sanderson and colleagues proposed a method to employ negative control outcomes to detect population stratification [28]. They suggest using phenotypes that are determined before the exposure and outcome of interest to make sure there is no causal link between the negative control outcome and the exposure of interest, and to explicitly consider population stratification when selecting a negative control. Examples provided are hair colour, eye colour, and skin tone. They describe two applied examples, including an MR on the effect of body mass index on coronary heart disease.  An alternative method is to use negative control populations, defined as a group of individuals that is similar to the population of interest except that there causal link between the exposure and outcome of interest is absent [29]. For example, when studying the effect of alcohol consumption or smoking on health outcomes, a group of people that abstain from such behaviour regardless of their genetic profile could serve as a negative control population. Piccininni and Stensrud provide a formal definition of negative control populations and also discuss their application outside of MR [29]. |
| Methodological | Negative controls have also been used to address concerns regarding study designs or analytical approaches. This underscores the potential of negative controls to not only detect or correct for bias in individual studies, but also strengthen causal inference in observational data across the board. The examples listed here are meant to illustrate how negative controls can be used for methodological research, and are not exhaustive.  Mendelian Randomisation (MR): negative controls have been used to evaluate the validity of the non-linear MR method used to evaluate J-shaped associations [30] and the doubly-ranked MR method developed to extend causal inferences made based on population-wide data to population subgroups [31]. Wade and colleagues demonstrated that the non-linear MR method was biased by showing an association between body mass index and the negative control outcome sex [30]. Hamilton and colleagues did the same for the doubly-ranked MR method by showing associations between both body mass index and Vitamin D exposure and the negative control outcomes age and sex. They also found biased results for a positive control (LDL cholesterol and myocardial infarction) [31]. This led to the retraction of a highly influential MR analysis on the effect of vitamin D on mortality [30,31].  Confounding by indication: negative control outcomes have been used to demonstrate confounding by indication in observational studies examining the effect of a novel lipid-lowering therapy, PCSK9i, on cardiovascular events [32]. The authors show that the beneficial effect of PCSK9i demonstrated in clinical trials may not be replicated in observational studies due to imbalances in health status (e.g., frailty) and health-seeking behaviour between those prescribed a PCSK9i and those prescribed other lipid-lowering therapies.  Selection bias in case-control studies: negative control exposures have been used to demonstrate selection bias due to survivor bias (selection on exposed cases) in a case-control study on concomitant treatment with statins and vitamin K antagonists and bleeding risk [33]. The authors show that the strongly beneficial effect of concomitant statin use was also seen for blood group, suggesting that exposed cases (those who took statins and had a bleeding event) were less likely to be included in the study because they had already died or were severely ill, which led to the beneficial effect observed.  Empirical evaluation of “fitness-for-purpose”: recently, negative control methods have been extended to empirical evaluation or benchmarking of study designs [34,35] and *a priori* testing of data and design suitability to answer a specific research question [36]. Schuemie and colleagues have used large selections of negative control outcomes and synthetic positive controls to empirically evaluate the performance of various epidemiological designs, using metrics like the coverage, mean precision and mean squared error, and Type I and Type II error. They generally found that self-controlled methods perform best across most scenarios [34,35]. Based on a narrative review, Levintow and colleagues provide a flowchart guiding how to incorporate negative control outcomes in study design and analysis, which includes using negative control outcomes to perform a *go/no-go* decision based on pre-specified criteria regarding the degree of bias or confounding that is unacceptable [36]. |

**References**

1.	Acton EK, Willis AW, Hennessy S. Core concepts in pharmacoepidemiology: Key biases arising in pharmacoepidemiologic studies. *Pharmacoepidemiol Drug Saf*. Jan 2023;32(1):9-18. doi:10.1002/pds.5547

2.	Zafari Z, Park JE, Shah CH, et al. The State of Use and Utility of Negative Controls in Pharmacoepidemiologic Studies. *Am J Epidemiol*. Feb 5 2024;193(3):426-453. doi:10.1093/aje/kwad201

3.	D'Arcy M, Stürmer T, Lund JL. The importance and implications of comparator selection in pharmacoepidemiologic research. *Curr Epidemiol Rep*. Sep 2018;5(3):272-283. doi:10.1007/s40471-018-0155-y

4.	Suissa S, Ernst P. Bias in observational study of the effectiveness of nasal corticosteroids in asthma. *J Allergy Clin Immunol*. Apr 2005;115(4):714-9. doi:10.1016/j.jaci.2004.12.1118

5.	Bots SH, Belitser S, Groenwold RHH, et al. Applying two approaches to detect unmeasured confounding due to time-varying variables in a self-controlled risk interval design evaluating COVID-19 vaccine safety signals, using myocarditis as a case example. *American Journal of Epidemiology*. 2024;doi:10.1093/aje/kwae172

6.	Huitfeldt A, Hernan MA, Kalager M, Robins JM. Comparative Effectiveness Research Using Observational Data: Active Comparators to Emulate Target Trials with Inactive Comparators. *EGEMS (Wash DC)*. 2016;4(1):1234. doi:10.13063/2327-9214.1234

7.	Londhe AA, Holy CE, Weaver J, Fonseca S, Villasis-Keever A, Fife D. Risk of retinal detachment and exposure to fluoroquinolones, common antibiotics, and febrile illness using a self-controlled case series study design: Retrospective analyses of three large healthcare databases in the US. *PLoS One*. 2022;17(10):e0275796. doi:10.1371/journal.pone.0275796

8.	Prasad V, Jena AB. Prespecified Falsification End Points: Can They Validate True Observational Associations? *JAMA*. 2013;309(3):241-242. doi:10.1001/jama.2012.96867

9.	Mohyuddin GR, Prasad V. Detecting Selection Bias in Observational Studies—When Interventions Work Too Fast. *JAMA Internal Medicine*. 2023;183(9):897-898. doi:10.1001/jamainternmed.2023.2067

10.	Groenwold RH. Falsification end points for observational studies. *Jama*. May 1 2013;309(17):1769-70. doi:10.1001/jama.2013.3089

11.	Hamer M, Bauman A, Bell JA, Stamatakis E. Examining associations between physical activity and cardiovascular mortality using negative control outcomes. *Int J Epidemiol*. Aug 1 2019;48(4):1161-1166. doi:10.1093/ije/dyy272

12.	Zaadstra BM, Chorus AM, van Buuren S, Kalsbeek H, van Noort JM. Selective association of multiple sclerosis with infectious mononucleosis. *Mult Scler*. Apr 2008;14(3):307-13. doi:10.1177/1352458507084265

13.	Rogawski McQuade ET, Liu J, Kang G, et al. Protection From Natural Immunity Against Enteric Infections and Etiology-Specific Diarrhea in a Longitudinal Birth Cohort. *J Infect Dis*. Nov 9 2020;222(11):1858-1868. doi:10.1093/infdis/jiaa031

14.	Arnold BF, Ercumen A. Negative Control Outcomes: A Tool to Detect Bias in Randomized Trials. *Jama*. Dec 27 2016;316(24):2597-2598. doi:10.1001/jama.2016.17700

15.	Murray EJ. Editorial: Demystifying the Placebo Effect. *American Journal of Epidemiology*. 2020;190(1):2-9. doi:10.1093/aje/kwaa162

16.	Wanis KN, Sarvet AL. Placebo Adherence as a Negative Control Exposure. *Epidemiology*. 2024;35(5)

17.	Flanders WD, Strickland MJ, Klein M. A New Method for Partial Correction of Residual Confounding in Time-Series and Other Observational Studies. *American Journal of Epidemiology*. 2017;185(10):941-949. doi:10.1093/aje/kwx013

18.	Sofer T, Richardson DB, Colicino E, Schwartz J, Tchetgen Tchetgen EJ. On negative outcome control of unobserved confounding as a generalization of difference-in-differences. *Stat Sci*. 2016;31(3):348-361. doi:10.1214/16-sts558

19.	Yu Y, Li H, Sun X, et al. Identification and Estimation of Causal Effects Using a Negative-Control Exposure in Time-Series Studies With Applications to Environmental Epidemiology. *Am J Epidemiol*. Feb 1 2021;190(3):468-476. doi:10.1093/aje/kwaa172

20.	Pearce N, Vandenbroucke JP, Lawlor DA. Causal Inference in Environmental Epidemiology: Old and New Approaches. *Epidemiology*. May 2019;30(3):311-316. doi:10.1097/ede.0000000000000987

21.	Wang Y, Danesh Yazdi M, Wei Y, Schwartz JD. Air pollution below US regulatory standards and cardiovascular diseases using a double negative control approach. *Nat Commun*. Sep 30 2024;15(1):8451. doi:10.1038/s41467-024-52117-8

22.	Steenland K, Schubauer-Berigan MK, Vermeulen R, et al. Risk of Bias Assessments and Evidence Syntheses for Observational Epidemiologic Studies of Environmental and Occupational Exposures: Strengths and Limitations. *Environmental Health Perspectives*. 2020;128(9):095002. doi:doi:10.1289/EHP6980

23.	Richardson DB, Keil AP, Tchetgen Tchetgen E, Cooper G. Negative Control Outcomes and the Analysis of Standardized Mortality Ratios. *Epidemiology*. Sep 2015;26(5):727-32. doi:10.1097/ede.0000000000000353

24.	Brew BK, Gong T, Williams DM, Larsson H, Almqvist C. Using fathers as a negative control exposure to test the Developmental Origins of Health and Disease Hypothesis: A case study on maternal distress and offspring asthma using Swedish register data. *Scand J Public Health*. Jul 2017;45(17_suppl):36-40. doi:10.1177/1403494817702324

25.	Smith GD. Negative control exposures in epidemiologic studies. *Epidemiology*. Mar 2012;23(2):350-1; author reply 351-2. doi:10.1097/EDE.0b013e318245912c

26.	Haan E, Sallis HM, Zuccolo L, et al. Prenatal smoking, alcohol and caffeine exposure and maternal-reported attention deficit hyperactivity disorder symptoms in childhood: triangulation of evidence using negative control and polygenic risk score analyses. *Addiction*. May 2022;117(5):1458-1471. doi:10.1111/add.15746

27.	Brynge M, Sjöqvist H, Gardner RM, Lee BK, Dalman C, Karlsson H. Maternal infection during pregnancy and likelihood of autism and intellectual disability in children in Sweden: a negative control and sibling comparison cohort study. *Lancet Psychiatry*. Oct 2022;9(10):782-791. doi:10.1016/s2215-0366(22)00264-4

28.	Sanderson E, Richardson TG, Hemani G, Davey Smith G. The use of negative control outcomes in Mendelian randomization to detect potential population stratification. *Int J Epidemiol*. Aug 30 2021;50(4):1350-1361. doi:10.1093/ije/dyaa288

29.	Piccininni M, Stensrud MJ. Using Negative Control Populations to Assess Unmeasured Confounding and Direct Effects. *Epidemiology*. 2024;35(3):313-319. doi:10.1097/ede.0000000000001724

30.	Wade KH, Hamilton FW, Carslake D, Sattar N, Davey Smith G, Timpson NJ. Challenges in undertaking nonlinear Mendelian randomization. *Obesity (Silver Spring)*. Dec 2023;31(12):2887-2890. doi:10.1002/oby.23927

31.	Hamilton FW, Hughes DA, Spiller W, Tilling K, Davey Smith G. Non-linear Mendelian randomization: detection of biases using negative controls with a focus on BMI, Vitamin D and LDL cholesterol. *Eur J Epidemiol*. May 2024;39(5):451-465. doi:10.1007/s10654-024-01113-9

32.	Levintow SN, Orroth KK, Breskin A, et al. Use of negative control outcomes to assess the comparability of patients initiating lipid-lowering therapies. *Pharmacoepidemiol Drug Saf*. Apr 2022;31(4):383-392. doi:10.1002/pds.5396

33.	van Rein N, Cannegieter SC, Rosendaal FR, Reitsma PH, Lijfering WM. Suspected survivor bias in case-control studies: stratify on survival time and use a negative control. *J Clin Epidemiol*. Feb 2014;67(2):232-5. doi:10.1016/j.jclinepi.2013.05.011

34.	Schuemie MJ, Arshad F, Pratt N, et al. Vaccine Safety Surveillance Using Routinely Collected Healthcare Data-An Empirical Evaluation of Epidemiological Designs. *Front Pharmacol*. 2022;13:893484. doi:10.3389/fphar.2022.893484

35.	Schuemie MJ, Cepeda MS, Suchard MA, et al. How Confident Are We about Observational Findings in Healthcare: A Benchmark Study. *Harv Data Sci Rev*. 2020;2(1)doi:10.1162/99608f92.147cc28e

36.	Levintow SN, Nielson CM, Hernandez RK, et al. Pragmatic considerations for negative control outcome studies to guide non-randomized comparative analyses: A narrative review. *Pharmacoepidemiology and Drug Safety*. 2023;32(6):599-606. doi:<https://doi.org/10.1002/pds.5623>
