# Supplementary material for: Negative controls and how to use them: a guidance paper
Source: Int J Epidemiol. 2026 Aug 29;55(5):dyag163. doi: 10.1093/ije/dyag163 (PMC13525841; doi:10.1093/ije/dyag163)
Supplement: Supplementary file 1 [file dyag163_supplementary_data.zip › ije-2025-06-1048-File004.doc]

**Supplementary Table S2**	**STROBE-style checklist of items that should be included in reports of negative control analyses**

**Part 1	Specifying the selection and analysis of negative controls in the analysis protocol**

|  | Item | Recommendation |
| --- | --- | --- |
| *Lay the groundwork* |  |  |
| Aims/objectives of the negative control analysis | 1 | (a) Outline the anticipated source(s) of bias and/or error to be addressed: selection bias, information bias, confounding, a combination, or type I error. |
|  |  | (b) Specify the purpose of the negative control analysis: bias detection, bias correction of estimate, calibration of p-value or confidence interval, or a combination. |
|  |  | (c) Describe the hypothesised sources of bias shared with the variable of interest (supported by literature and/or graphical displays), focussed on bias factors the negative control is meant to address. |
| Method used to identify negative controls | 2 | Specify the method used to identify potential negative controls, including any decision rules applied;   - Manually, based on literature review and expert assessment. - Data-driven, using existing screening tools or reference sets. - Literature-driven, using negative controls from existing studies examining the same association of interest where one or more negative controls were extensively evaluated. |
| List of potential negative controls | 3 | Provide a list of all potential negative controls (in the protocol or as appendix); all exposures and/or outcomes that are not causally associated with the outcome (NCE) or exposure (NCO) of interest. |
| *Assess feasibility and assumptions* | | |
| Practical concerns | 4 | Explain how the selection of potential negative controls was narrowed down based on what is feasible within the available data source(s) and chosen study design (practical concerns).   - If possible, avoid having to select a new study population for the negative control analysis (e.g., applying an NCO in a case-control design). - Select negative controls that are sufficiently prevalent in the data source to avoid power issues and structural non-positivity. - Describe steps taken to ensure measurement of negative controls is of sufficient quality (validity). |
| Concerns related to the goal/purpose of the analysis (point 1) | 5* | (a) Explain how the selection of potential negative controls was narrowed down based on relevant assumptions and requirements of the chosen purpose.   - Bias detection: negative controls should match the bias structure of the original exposure (NCE) or outcome (NCO). - Bias correction: negative control definition same as bias detection. Any bias correction method-specific assumptions should be met. - Calibration of p-values: negative controls should not be causally associated with the exposure (NCO) or outcome (NCE) under study. Requires a sufficient number of negative controls to estimate the underlying bias distribution. A (partially) shared bias structure with the exposure-outcome relation is not explicitly required, but will improve the effectiveness of the method. - Calibration of confidence intervals: assumptions and requirements for negative control selection are the same as calibration of p-values (above). Creation of positive controls is required and can be done by artificially increasing the incidence/prevalence of selected negative controls through predictive modelling^ψ^. |
|  |  | (b) Decide on the number and type of negative control (exposure/outcome/both) based on the goal/purpose of the analysis.   - Bias detection or bias correction of estimate: one negative control that closely matches the bias in the exposure-outcome relation, unless the chosen method explicitly requires more (e.g., the confounding bridge approach). - p-value or confidence interval calibration: a sufficient number of negative controls to allow construction of the underlying error distribution. |
| Quality concerns | 6 | Check for factors that would rule out certain approaches and explain how these influenced the negative control selection   - Bias detection is not of added value if a negative control that (sufficiently) shares the relevant bias structure cannot be identified. Consider alternative sensitivity analyses that do not use negative controls. - Bias correction of estimates is not reliable in the presence of (differential) measurement error. If you still want to apply negative controls, consider moving to the bias detection approach. - Calibration (p-value, confidence interval) is not reliable when the negative controls are subject to a different (higher/lower) extent of bias than the association of interest, such that the underlying systematic error distributions do not match. Consider using uncalibrated values instead. It is also not possible when you cannot identify sufficient negative controls to construct the systematic error distribution. - Consider your target audience and which approaches they are likely to understand and trust (face validity). |
| *Finalise the negative control selection* | | |
| List of negative controls | 7 | Provide a list of the final negative controls (in the protocol or as appendix). Can be combined with the list from point 3. |
| *Specify statistical analysis plan* | | |
| Statistical methods | 8 | (a) Method-specific assumptions and how these will be checked. |
|  |  | (b) Method-specific analysis procedures. |
| Criteria for the null effect | 9^#^ | Specify what is considered a null effect for the negative control analysis and how will this be interpreted.  Bias detection  *Based on statistical testing^¥^*   - The negative control test is not statistically significant, based on a pre-specified threshold, e.g. family-wise error rate. Authors should consider their specific case and support their threshold choice. - The confidence interval, with width pre-specified by a threshold (e.g., the family-wise error rate), should include the null.     *Based on effect size*   - The effect estimate and confidence interval of the negative control analysis should fall within certain bounds deemed to indicate a low risk of bias (for example, between 0.8 and 1.2)^§^. - Only effect sizes larger than those of the negative control analysis should be considered true effects.   Bias correction   - COCA: the crude and adjusted effect should not be statistically different (*see statistical testing above*). If this does not hold, there is unmeasured confounding and the adjusted estimate should be used. - Confounding bridge: solving the confounding bridge equation should lead to a null result (*see statistical testing above*). If this does not hold, there is unmeasured confounding and the adjusted estimate should be used.   P-value or 95% confidence interval calibration   - Acknowledge that this approach works under the strong assumption that the true risk estimate of any negative control is 1 and that any deviation from this signals the presence of bias |
| Power considerations | 10 | Include a power calculation for the negative control analysis, and specify the minimum amount of events required for the results of the negative control analysis to provide interpretable results given the criteria for the null effect specified under point 9. |

*Researchers may need to cycle between items 1-6 to make sure they choose a proper and valid approach before moving on to item 7.

^#^ Definitions based on statistical testing rely on sufficient sample size; an underpowered negative control analysis may meet the criteria but confer limited information regarding the presence or absence of bias. Existing literature does not discuss pre-specifying a null effect for bias correction and p-value/confidence interval calibration methods. It has been suggested to define a maximum degree of bias prior to running the negative control analysis [1]. If the negative control analysis suggests the degree of bias is higher than the pre-specified threshold, it might signal underlying issues in the analysis, like insufficient confounding control, that need to be addressed before continuing.

^¥^ Using p-values and confidence intervals to denote statistical significance in a binary fashion (“significant” vs “not significant”) has been critiqued [2]. We would recommend either combining this definition with an effect size-based definition of the null effect, or interpreting the results with appropriate nuance.

^§^ This suggestion follows the reasoning for defining non-inferiority margins; see [3] for more information on this topic.

^ψ^ Following the approach outlined by [4]

**Part 2	Reporting a negative control analysis in the manuscript**

|  | **Item** | **Recommendation** |
| --- | --- | --- |
| **Title and abstract** | 1 | The use of and, if space allows, outcome of the negative control analysis. |
| **Methods*** |  |  |
| Bias | 2 | (a) Anticipated issue the analysis should address: e.g., selection bias, information bias, confounding, a combination, or increased type I error. |
|  |  | (b) Goal of the negative control analysis: bias detection, bias correction of estimate, calibration of p-value or confidence interval. |
| Variables | 3 | Negative control selection process, ideally based on a previously published protocol (*see Part 1 of the checklist above*).   - Rationale for final selection of negative control(s). - Hypothesised bias structure of variable of interest (including arguments), focussed on bias factors the negative control analysis is meant to address. - Decision rules for automated selection, if used. |
| Study size | 4 | Power considerations for the negative control analysis and whether the power was sufficient to provide interpretable results. |
| Statistical methods | 5 | (a) Negative control method-specific assumptions and (whenever possible) whether these are likely to have been met. |
|  |  | (b) Negative control method-specific analysis steps. |
|  |  | (c) Criteria for null effect; what is considered a null effect for the negative control analysis and how will this be interpreted. |
| **Results** | 6 |  |
| Negative control analyses* |  | Depending on the negative control analysis chosen:   - Bias detection: risk estimate(s) and 95% confidence interval (s) for the negative control(s). - Bias correction: both the unadjusted and adjusted risk estimate and 95% confidence interval for the association of interest. - Calibration of p-values or confidence intervals: both the uncalibrated and calibrated p-values or confidence intervals for the association of interest.   - Consider adding overview plots, for example a funnel plot with all negative control estimates centred around the expected null effect, for transparency.   Risk estimates can be reported as numbers in-text, in a Table, or as a Figure (forest plots are commonly used). When opting for a Figure, report the exact risk estimates and 95% confidence interval in the Supplementary Files. |
| **Discussion** | 7 |  |
| Interpretation |  | A paragraph interpreting the study findings in light of the negative control findings, including any limitations of the negative control analysis and how these may have affected the negative control analysis findings. |

*We propose that authors include a ‘negative control’ paragraph in their methods and results sections to report all relevant points listed above. Good examples can be found here [5,6].

References
